# A mild stressor induces short-term anxiety and long-term phenotypic changes in trauma-related behavior in female rats

**DOI:** 10.3389/fnbeh.2023.1231563

**Published:** 2023-09-04

**Authors:** Khadijah Shanazz, Rebecca Nalloor, Almira Vazdarjanova

**Affiliations:** ^1^VA Research Service, Charlie Norwood VA Medical Center, Augusta, GA, United States; ^2^Department of Neuroscience and Regenerative Medicine, Medical College of Georgia at Augusta University, Augusta, GA, United States; ^3^Department of Pharmacology and Toxicology, Medical College of Georgia at Augusta University, Augusta, GA, United States

**Keywords:** PTSD, avoidance extinction, stress history, LDOF, contextual fear conditioning, fear extinction

## Abstract

**Introduction:**

Anxiety and anxiety-influenced disorders are sexually dimorphic with women being disproportionately affected compared to men. Given the increased prevalence in women and the documented differences in anxiety and trauma behavior between male and female rats this paper sought to examine the link between stress, anxiety, and fear learning and extinction in female rats. We tested the hypothesis that a mild stressor will induce short-and long-term increases in anxiety and produce long term effects on subsequent fear learning and extinction behavior.

**Methods:**

We induced anxiety in female Sprague– Dawley rats with a short (3 min) exposure to a ball of cat hair infused with 150 μl of cat urine (mild stressor) that elicits innate fear but does not cause fear conditioning. The control group was exposed to fake cat hair. Anxiety was assessed in the Light-Dark Open Field (LDOF) or Elevated Plus Maze (EPM) before, immediately after and 4 days after stimulus exposure. Two weeks later, all animals were subject to Contextual Fear Conditioning (CFC) in the Shock Arm of a Y-maze, blocked off from the rest of the maze. Memory and fear extinction (learning of safety) was assessed in the following four days by placing each rat in one of the Safe Arms and measuring avoidance extinction (time spent and number of entries in the Shock Arm).

**Results:**

Cat hair exposure induced changes in anxiety-like behavior in the short-term that appeared resolved 4 days later. However, the cat-hair exposed rats had long-term (2 weeks) phenotypic changes expressed as altered exploratory behavior in an emotionally neutral novel place. Fear learning and extinction were not impaired. Yet, using avoidance extinction, we demonstrated that the phenotypic difference induced by the mild stressor could be documented and dissociated from learning and memory.

**Discussion:**

These findings demonstrate that the history of stress, even mild stress, has subtle long-term effects on behavior even when short-term anxiety appears resolved.

## Background

Anxiety is a state of fear, worry, or unease that is characterized by physical symptoms such as increased heart rate, sweating, trembling, and difficulty concentrating (American Psychiatric Association, 2013). Anxiety is not a uniform concept and is separated into two broad categories: state anxiety, anticipation of a negative experience with a stimulus, and trait anxiety, a characteristic of the individual (Steimer, 2011; Saviola et al., 2020) with varying symptomology and severity (Bandelow et al., 2017). Anxiety disorders such as generalized anxiety disorder, panic disorder, phobias, etc., by themselves affect nearly 34% of the population (Bandelow and Michaelis, 2015). Anxiety is a component of many psychiatric disorders, such as depression, obsessive compulsive disorder, and post-traumatic stress disorder (PTSD; Craske et al., 2017) and is sexually dimorphic with women typically reporting higher levels of anxiety than men (Maeng and Milad, 2015; Jalnapurkar et al., 2018; Bangasser and Cuarenta, 2021).

Another aspect of anxiety is that its relationship with stress is bidirectional such that stress can cause anxiety and anxiety can cause stress (Kessler and Greenberg, 2002; Li and Goldsmith, 2012; Shamsuddin et al., 2013). Stress is considered “a strain or a pressure of a constraining or impelling nature” (Izquierdo et al., 2016) and a stressor is a stimulus that has such an effect. The effect of a stressor then varies based on perceived intensity and tends to range from mild to traumatic. We delineate between these two by defining a mild stressor as one that induces stress responses such as HPA axis activation but does not induce fear conditioning whereas a traumatic stressor does induce fear conditioning. Conditioned fear responses tend to be freezing and/or avoidance behavior to stimuli previously associated with the stressor that is above baseline (Shanazz et al., 2022). It is imperative to understand the relationship between anxiety and stress in the short and long term, especially given the high prevalence of anxiety and stress caused by the COVID-19 pandemic across the world (Al Omari et al., 2020; Kamal and Othman, 2020; Lakhan et al., 2020; Alnazly et al., 2021; Shah et al., 2021).

The relationship between anxiety and stress can be extrapolated to PTSD. PTSD and anxiety are both characterized by intrusive and persistent thoughts and memories, difficulty controlling emotions, and avoidance of situations associated with the trauma or negative stimulus. Moreover, anxiety before trauma has been shown to be associated with a higher rate of developing PTSD (Mason et al., 2002; Cameron et al., 2006; Sareen, 2014) making anxiety a risk factor for developing PTSD (Alexander et al., 2020). After trauma, PTSD shares a high comorbidity with anxiety (Spinhoven et al., 2014; Gagne et al., 2018), so much so that it was previously classified as an anxiety disorder in the DSM-IV (American Psychiatric Association, 2000). Like anxiety, PTSD is sexually dimorphic with women being affected more than men (Kessler et al., 1995; Christiansen and Elklit, 2012; Gamwell et al., 2015). The relationship between stress, anxiety, and response to trauma in female rats is the focus of the study presented here.

Similar to humans, the relationship between PTSD-like responses and anxiety-like behavior is documented in rats. Rats exposed to traumatic events such as foot-shock display more anxiety-like behavior on the elevated plus maze (Davis, 1990; Korte et al., 1999; Mechiel Korte and De Boer, 2003). Conversely, rats that display high anxiety-like behavior prior to trauma exposure have an exaggerated PTSD-like phenotype after trauma exposure (Nalloor et al., 2011). Thus, the relationship between anxiety and PTSD is clearly documented in both humans and rats such that anxiety before trauma contributes to PTSD outcomes and PTSD contributes to anxiety outcomes. We tested the hypothesis that a stressor will exacerbate both short-and long-term anxiety-like behavior and subsequent trauma response in female rats.

## Methods

### Animal handling

**Figure 1:**
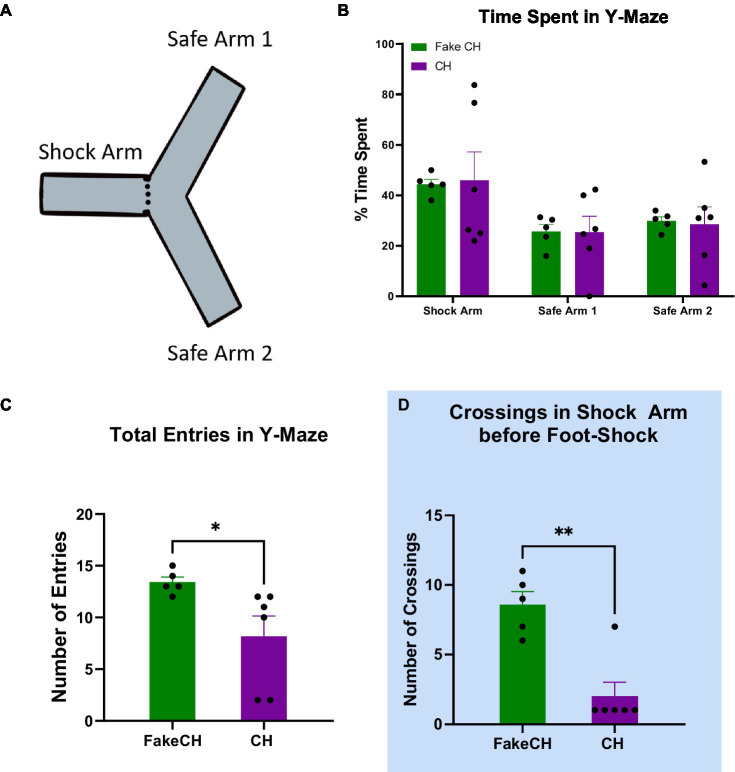
Novel exploration 2 weeks after cat hair exposure or fake cat hair exposure. **(A)** Schematic of Y-Maze. **(B)** Time spent in the Shock Arm, Safe Arm 1, and Safe Arm 2 of the Y-maze during habituation. **(C)** Total entries in the Y-Maze during habituation. **(D)** Crossings in the Shock Arm before foot-shock the day after Y-Maze habituation (FakeCH: Fake cat hair; CH: Cat hair). Unpaired two-tailed *t*-tests ^*^p < 0.05, ^**^p < 0.01.

**Figure 2:**
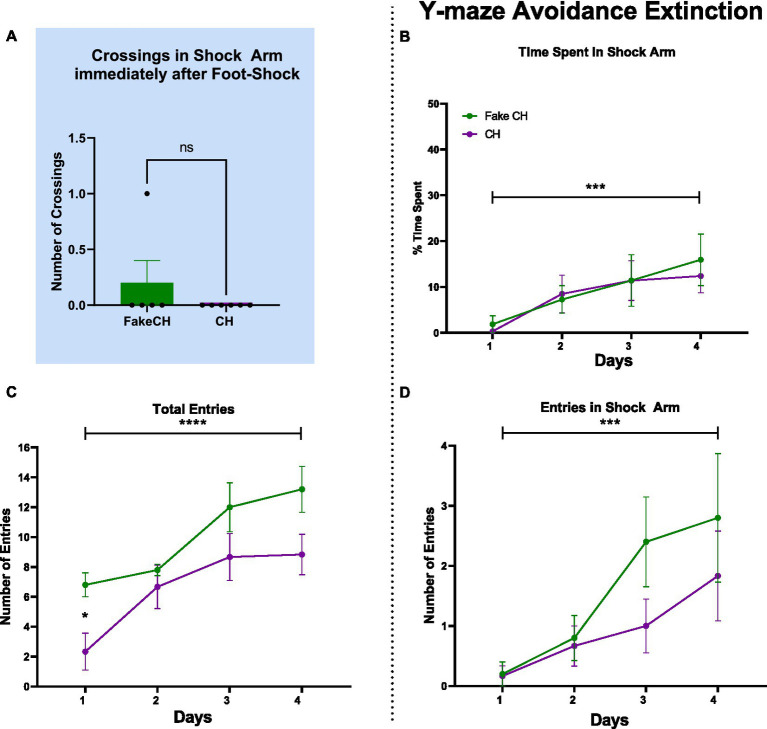
Post-shock behavior and avoidance extinction in the Y-maze. **(A)** Crossings in the Shock Arm after foot-shock, Unpaired two-tailed *t*-test. **(B)** Time spent in the Shock Arm during avoidance extinction. **(C)** Total entries in the Y-maze during avoidance extinction. **(D)** Entries into the Shock Arm during avoidance extinction (FakeCH: Fake cat hair; CH: Cat hair). Repeated measures ANOVA ^***^p < 0.001, ^****^p < 0.0001, Repeated measures ANOVA *post-hoc* ^*^p < 0.05, and ns, not significant.

Young adult (2 months old) female (175–200 g) Sprague–Dawley rats (Charles River Laboratories Inc., MA, United States) were housed in pairs on a 12 h light/dark cycle (lights on at 7:00 am) with *ad lib* food and water. Experiments were conducted during the light phase between 8 am and 5 pm. Three days after arrival, rats were handled for 2–3 min for three consecutive days prior to behavioral testing. Both Fake cat hair (FakeCH) and Cat Hair (CH) groups had six animals each except for Figures 1, 2 where one animal was excluded from the FakeCH group due to shock equipment malfunction during foot-shock delivery. Animals were allowed to cycle naturally during experiments and estrus phase was not analyzed. All behavioral procedures were approved by the Institutional Animal Care and Use Committee (IACUC) at the Charlie Norwood VA Medical Center (CNVAMC).

### Behavioral tests

All behavior was recorded for later analysis. Scoring was done by observers “blinded” to the experimental assignment of each animal. Behavioral testing was done in the same large room (24′ × 15′) on different days with different tests set up in different parts of the room such that extra maze cues were sufficiently different.

### Experimental design

**Figure 3:**
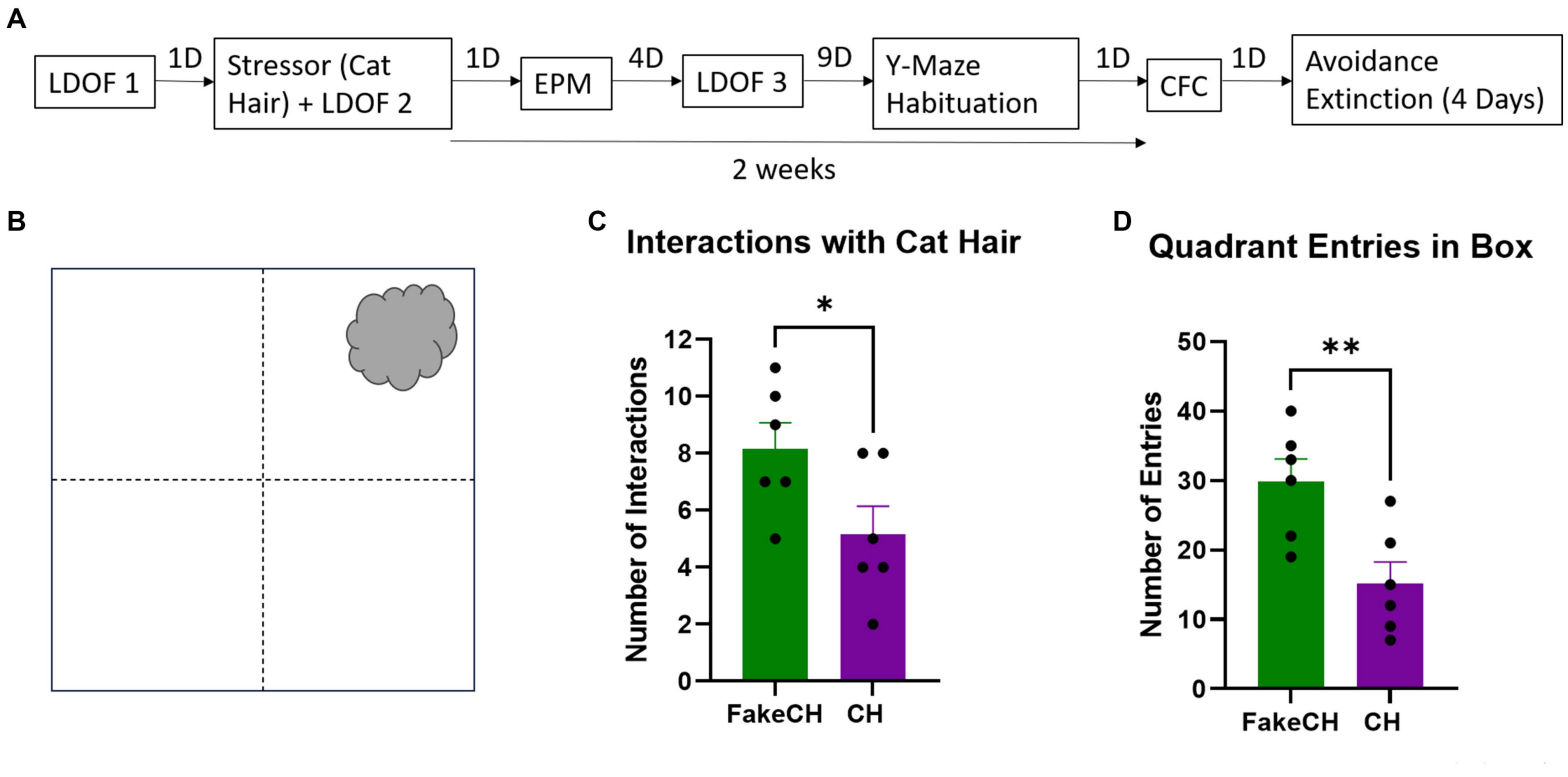
Cat hair is a mild stressor. **(A)** Experimental design: LDOF, Light–dark open field; EPM, Elevated plus maze; CFC, Contextual fear conditioning; and D, Days. **(B)** Schematic of cat hair box. **(C)** Number of interactions with Fake cat hair and cat hair. **(D)** Number of entries into quadrants of the box during exposure (FakeCH: Fake cat hair; CH: Cat hair). Unpaired two-tailed *t*-tests, ^*^p < 0.05, ^**^p < 0.01.

Animals were first tested on the Light–Dark Open Field (LDOF) to assess baseline locomotion and anxiety. A day later they were exposed to a stressor (cat hair) and tested in the LDOF 10–15 min later. The next day anxiety-like behavior was measured in the Elevated Plus Maze (EPM). Four days later anxiety was assessed again in the LDOF. Two weeks after cat hair exposure animals were habituated to the Y-Maze and experienced Contextual Fear Conditioning (CFC) followed by Avoidance Extinction in the Y-maze described in detail in Shanazz et al. (2022) (Figure 3A).

### Light–dark open field

**Figure 4:**
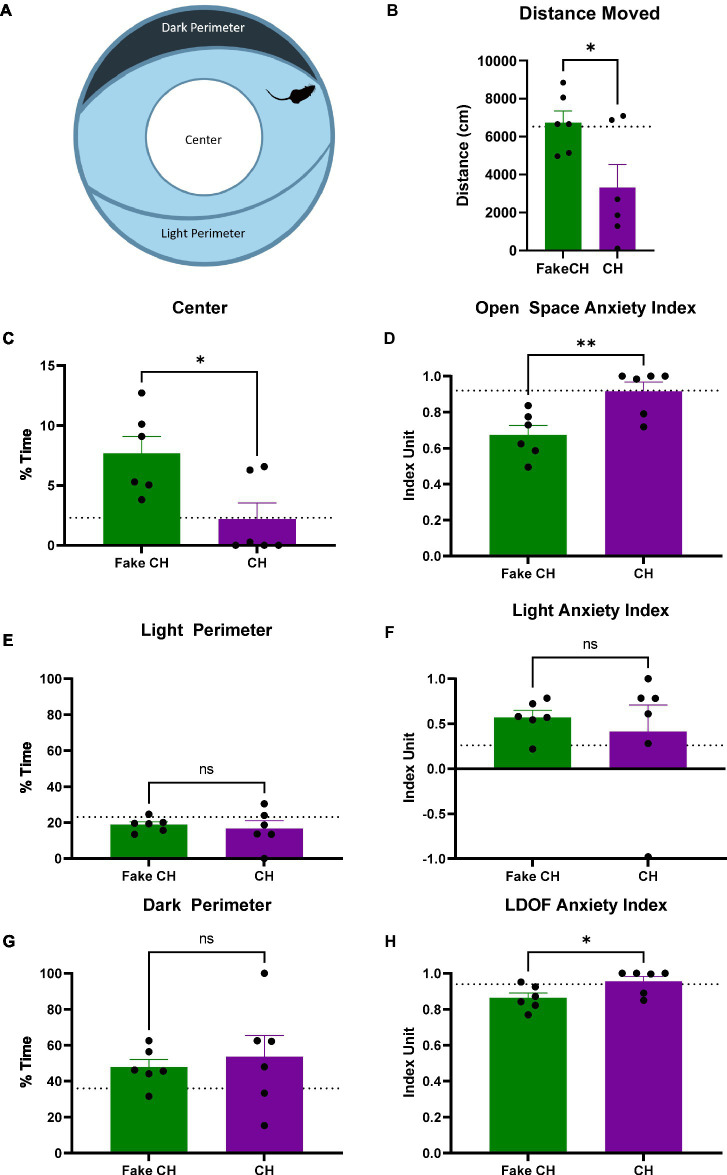
Anxiety-like behavior in the LDOF immediately after cat hair or fake cat hair exposure. **(A)** Schematic of Light–Dark Open Field (LDOF); **(B)** Distance moved over the session; **(C)** % Time spent in the center of the LDOF; **(D)** Open space anxiety index; **(E)** % Time spent in the light perimeter; **(F)** Light anxiety index; **(G)** % Time spent in the dark perimeter; **(H)** LDOF anxiety index (FakeCH: Fake cat hair; CH: Cat hair). Dashed lines represent the average of all animals on the initial test (LDOF 1). Unpaired two-tailed *t*-tests, ^*^p < 0.05, ^**^p < 0.01, and ns, not significant.

Animals were tested for anxiety-like behavior in the LDOF for 10 min. The LDOF (Figure 4A) is a circular arena measuring 142 cm diameter × 60 cm height with floodlights positioned on the floor to cast a shadow (called the Dark Perimeter) on the maze that covers approximately 20% of the arena. The delta lux between the Light and Dark perimeter was 40 which we have previously established as above the threshold for detecting anxiety to light (Shanazz et al., 2021). Behavior was analyzed with Noldus Ethovision XT 14 software (Noldus Information Technology Inc.). The apparatus was cleaned with dH2O between animals.

The LDOF arena has three areas (or zones):

- Center: 25% of the total arena area and a diameter of ½ of the total diameter of the arena.
- Dark Perimeter: 20% of the total arena; a shadowed crescent-shaped area adjacent to the wall.
- Light Perimeter: 20% of the total arena; a crescent-shaped area adjacent to the wall and opposite of the Dark Perimeter.

Three indices were used to interpret data from the LDOF (Shanazz et al., 2021):

1. The Light Anxiety Index = $\;1-\frac{\%TimeinLightPerimeter}{\%TimeinDarkPerimeter}$

This index quantifies the extent of light aversion such that higher numbers represent increased anxiety to light.

2. The Open Space Anxiety Index = $\;1-\frac{\frac{4}{5}\%TimeinCenter}{\%TimeinLightPerimeter}$

This index quantifies the extent of open space aversion such that higher numbers represent increased anxiety to open space.

3. The LDOF Anxiety Index = $\;1-\frac{\frac{4}{5}\%TimeinCenter}{\%TimeinDarkPerimeter}$

This index combines both anxiogenic components within the LDOF: light and open space, with the center representing high light and open space and the Dark Perimeter representing low light and sheltered space. The LDOF Anxiety Index allows for an integrated quantification of anxiety-like behavior such that higher numbers represent increased overall anxiety.

### Cat hair exposure

Animals were exposed for 3 min to a ball of cat hair (∼10 cm) obtained from a pathogen-free male cat and infused with ∼150 μl of cat urine in a 32 cm × 32 cm × 50 cm box (Figure 3B). We have previously shown that in male rats such exposure elicits unconditioned fear responses but does not induce CFC (Vazdarjanova et al., 2001; Nalloor et al., 2011, 2014) and thus is considered a mild stressor. A control group was exposed to Fake cat hair (FakeCH) of approximately the same size as the cat hair ball and comprised of clean pillow stuffing. Fresh cat hair and urine is acquired for each experiment session as standard lab practice. FakeCH animals were tested first followed by CH exposed animals to avoid any residual olfactory cues. Groups were run in separate boxes with the stimuli positioned in the upper right corner of the box (Figure 3B). The boxes were cleaned with dH2O between animals. To prevent lingering smell from the cat hair in the behavior room, the box was cleaned with 5% acetic acid after the last cat hair animal was tested. Cat hair interactions were scored when the animal’s head was within 1 cm of, or front paws were in physical contact with, the ball of cat hair. Fewer interactions with the stimulus indicate the animals perceive the stimulus as aversive. The box was virtually divided into four equal quadrants and the number of entries into each quadrant was scored when the body and base of the tail crossed the quadrant line of the box and were recorded as an indicator of overall activity.

### Elevated plus maze

**Figure 5:**
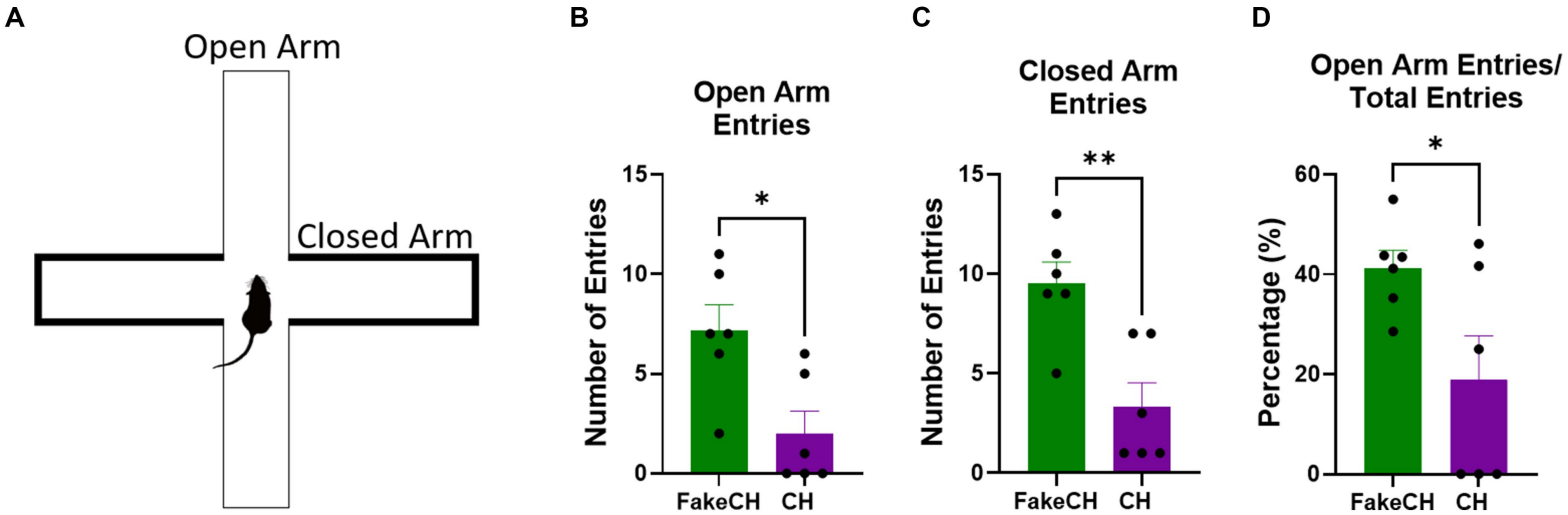
Anxiety-like behavior in the elevated plus maze (EPM) 1 day after cat hair or fake cat hair exposure. **(A)** Schematic of EPM. **(B)** Number of entries into the open arms. **(C)** Number of entries into the closed arms; **(D)** Ratio of open arm entries over total entries expressed as a percentage (FakeCH: Fake cat hair; CH: Cat hair). Unpaired two-tailed *t*-tests ^*^p < 0.05, ^**^p < 0.01.

We measured anxiety-like behavior on the EPM (Figure 5A) 1 day after Cat Hair exposure. The EPM is plus-shaped with four 50 cm × 12 cm arms, elevated 84 cm above the floor. Two opposite arms are surrounded by 46 cm tall opaque black walls on three sides, and the other two are open, except for a 1 cm high ledge (Kinder Scientific, San Diego, CA, United States). Each animal was introduced into the center area (10 cm × 10 cm) facing an Open Arm and allowed to explore freely for 5 min. An Arm Entry was scored when all four paws and the base of the animal’s tail entered an arm. More entries into the Open arms indicates lower anxiety-like behavior (Hogg, 1996). Closed arm entries are used as an indicator of activity, which may be influenced by anxiety state (Pellow et al., 1985; Gonzalez and File, 1997; Ramos et al., 1997). The apparatus was cleaned with dH2O between animals.

### Contextual fear conditioning training

The Y-maze apparatus consists of three identically shaped arms (50 cm × 20 cm × 12 cm), separated by 120° (Figure 1A) and covered with translucent Plexiglass lids. One arm of the Y-maze (henceforth referred to as Shock Arm) was fitted with stainless steel walls and floor plates separated by a 1 cm gap in the floor through which a foot-shock can be administered and a removable wall that allows this arm to be isolated from the other arms. Visual cues were distinct between arms. Rats were habituated to the Y-maze for 5 min the day before CFC training to assess whether they have a natural aversion/preference to any of the arms. Animals were started in the Shock Arm during habituation and Time Spent per arm and the number of Arm Entries were scored. The day after habituation, animals experienced CFC in the isolated Shock Arm of a Y-maze as previously described (Vazdarjanova and McGaugh, 1998). After 3 min of acclimation, animals received two foot-shocks (1 mA ac, 1 s) delivered at 30-s intervals. Ninety seconds after the second foot-shock, the rats were removed from the Shock Arm and returned to their home cages. Number of crossings is scored as a measure of locomotor activity and defined as all four paws and the base of the tail crossing over the midline of the Shock Arm. The apparatus is cleaned with 10% ethanol between animals.

### Avoidance fear extinction

The day after CFC, avoidance fear extinction was performed in the Y-maze as previously reported (Shanazz et al., 2022). Rats were reintroduced into one of the Safe Arms of the Y-maze (alternating arms each day) and allowed to explore all arms for 5 min per day for 4 consecutive days, without foot-shocks. Time Spent in each arm, and the Number of Arm Entries were scored. Avoidance Extinction is defined as more entries or more Time Spent in the Shock Arm over days of extinction. Freezing is not reported because we and others have shown that female rats tend to freeze very little during extinction (Mitchell et al., 2022; Shanazz et al., 2022).

### Statistics

Unpaired two-tailed *t*-tests were used to compare Fake cat hair (FakeCH) and Cat Hair (CH) groups on Cat Hair measures, EPM, LDOF measures, Y-maze habituation measures, and behavior during CFC. Mixed-Design repeated measures-ANOVA with condition factor “Cat Hair” repeated factor “Time” was used to compare groups on Avoidance Extinction measures. Uncorrected Fisher’s LSD *post hoc* was used to compare Day 1 of Avoidance Extinction and Total Entries in the Y-maze to assess 24 h memory of the CFC training. Differences were considered statistically significant at *p* < 0.05. Graphs are reported with standard error of the mean. Data was analyzed with StatView (SAS Institute, Cary, NC, United States) and visualized with PRISM (GraphPad Prism version 8.0.0 for Windows, GraphPad Software, San Diego, CA, United States).

## Results

### Cat hair exposure is a mild stressor

Animals exposed to cat hair had fewer interactions with the stimulus [Figure 3C, two-sample *t*(10) = 2.243, *p* = 0.0488] and made fewer Entries into all quadrants of the box [Figure 3D, two-sample *t*(10) = 3.259, *p* = 0.0086] compared to fake cat hair exposed animals suggesting that exposure to cat hair is stressful. However, it is not a traumatic stressor the way foot-shock is (see Supplementary Figure 1).

### Cat hair exposure increases anxiety-like behavior in the LDOF immediately after exposure

Prior to cat hair exposure, there were no differences between groups in and of the LDOF measures indicating that there were no inherent differences in anxiety [Distance moved: two-sample *t*(10) = 0.0850, *p* = 0.9340, %Time Spent in Center: two-sample *t*(10) = 0.3819, *p* = 0.7105, %Time Spent in the Dark/Light Perimeter: two-sample *t*(10) = 0.0364, *p* = 0.9717, Light Anxiety Index: two-sample *t*(10) = 0.1374, *p* = 0.8934, Open Space Anxiety Index: two-sample *t*(10) = 0.3567, *p* = 0.7288, and LDOF Anxiety Index: two-sample *t*(10) = 0.4500, *p* = 0.6625, Average shown as dashed lines on Figure 4 graphs].

Immediately after exposure, animals exposed to cat hair moved less in the LDOF compared to animals exposed to fake cat hair [Figure 4B, two-sample *t*(10) = 2.497, *p* = 0.0316]. Additionally, cat hair exposed animals spent less time in the Center of the LDOF [Figure 4C, two-sample *t*(10) = 2.807, *p* = 0.0186] but there was no difference in time spent in the Light or Dark Perimeter [Figures 4E,G, two-sample *t*(10) = 0.4650, *p* = 0.6519]. Reflective of this, Cat Hair exposed animals had more anxiety to open space as their Open Space Anxiety Index was greater than that of Fake Cat Hair exposed animals [Figure 4D, two-sample *t*(10) = 3.284, *p* = 0.0082] while there was no difference in their Light Anxiety Index [Figure 4F, two-sample *t*(10) = 0.5129, *p* = 0.6192]. The difference in their open space anxiety was great enough to generate a difference in the combined LDOF Anxiety Index [Figure 4H, two-sample *t*(10) = 2.366, *p* = 0.0396].

### Cat hair exposure increases anxiety in the short term

One day after cat hair exposure we measured anxiety-like behavior on the EPM. Animals exposed to Cat Hair made fewer entries into the Open Arms [Figure 5B, two-sample *t*(10) = 3.003, *p* = 0.0133] indicating more anxiety-like behavior in these animals. In addition Cat hair exposed animals also had fewer Closed Arms entries [Figure 5C, two-sample *t*(10) = 3.804, *p* = 0.0035] indicating lower overall activity. Despite overall suppressed activity, Entries into the Open Arms were still proportionally lower in the Cat Hair group [Figure 5D, two-sample *t*(10) = 2.334, *p* = 0.0418] indicating that anxiety from the cat hair exposure persists for at least 24 h after exposure.

### Anxiety-like behavior from cat hair exposure is diminished by day 4 in the LDOF

**Figure 6:**
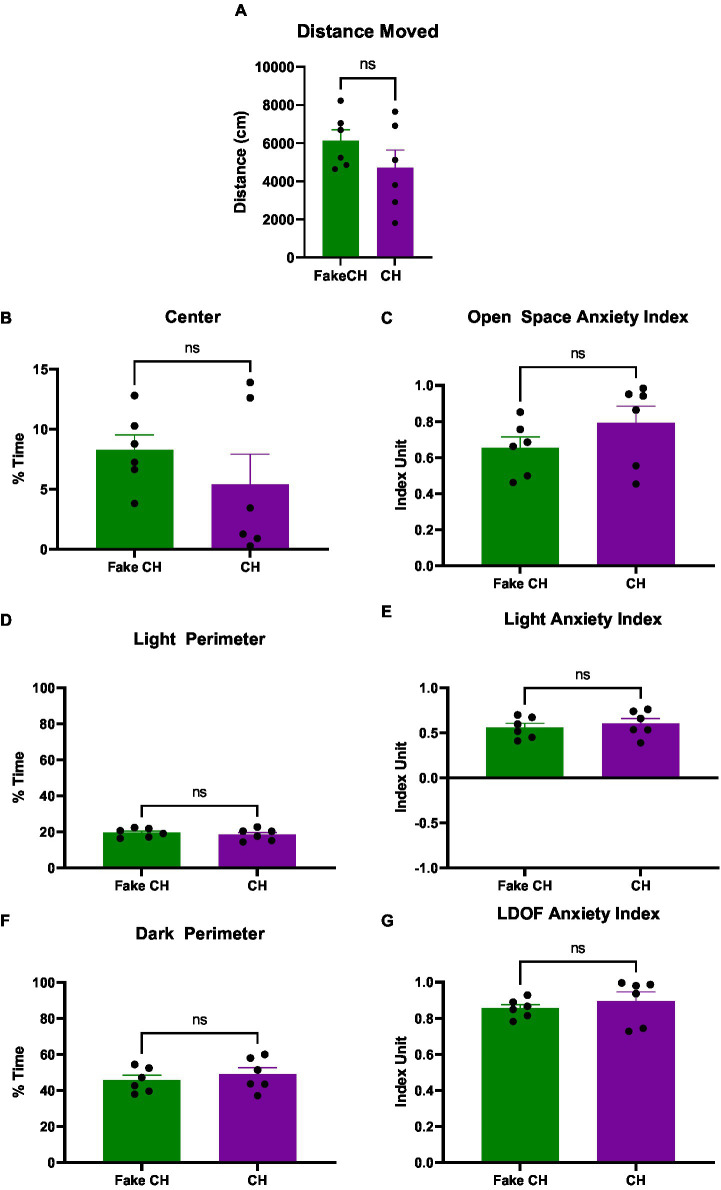
Anxiety-like behavior in the LDOF 4 days after cat hair exposure or fake cat hair exposure. **(A)** Distance moved over the session; **(B)** % Time spent in the center of the LDOF; **(C)** Open space anxiety index; **(D)** % Time spent in the light perimeter; **(E)** Light anxiety index; **(F)** % Time spent in the dark perimeter; **(G)** LDOF anxiety index (FakeCH: Fake cat hair; CH: Cat hair). Unpaired two-tailed *t*-tests. ns, not significant.

Four days after Cat Hair exposure, no differences were detectable between Cat Hair and Fake Cat Hair exposed animals in any of the measures of the LDOF [Figures 6A–G; A: two-sample *t*(10) = 1.284, *p* = 0.2282, B: two-sample *t*(10) = 1.011, *p* = 0.3357, C: two-sample *t*(10) = 1.247, *p* = 0.2407, D&F: two-sample *t*(10) = 0.7069, *p* = 0.4958, E: two-sample *t*(10) = 0.5993, *p* = 0.5623, and G: two-sample *t*(10) = 0.7325, *p* = 0.4807] indicating that the effect of cat hair exposure on anxiety measures persists for less than 4 days.

### Novel exploration is disrupted in cat hair exposed animals in the long-term

Two weeks after cat hair exposure, rats were habituated to the Y-maze by allowing them to explore freely—a novel exploration event before foot shock. There was no difference between groups in Time Spent in either of the safe arms [Safe Arm 1: two-sample *t*(10) = 0.0391, *p* = 0.9697; Safe Arm 2: two-sample *t*(10) = 0.1701, *p* = 0.8687] or the Shock Arm [two-sample *t*(10) = 0.1277, *p* = 0.9012; Figure 1B] indicating that there was no natural aversion to the Shock Arm. Note that during habituation, rats were started in the Shock Arm. Therefore, more time spent in this arm compared to others is expected. Cat Hair exposed animals had significantly fewer Total Entries compared to Fake Cat Hair [Figure 1C, two-sample *t*(10) = 2.347, *p* = 0.0435] demonstrating that their exploratory movement was suppressed. This is corroborated by significantly fewer Crossings in the Cat Hair group when they are confined to the Shock Arm the next day before the foot-shock was delivered [Figure 1D, two-sample *t*(10) = 4.759, *p* = 0.0010].

Although time spent in each arm was not different, the presence of a difference in exploration suggests that behavior was disrupted even 2 weeks after cat hair exposure.

### Cat hair exposure does not affect fear learning, but it does affect behavioral expression strategy after trauma

Despite differences in overall activity mentioned above, immediately after foot-shock, both Fake cat hair and Cat Hair exposed animals showed no difference in number of crossings in the shock arm [Figure 2A, two-sample *t*(10) = 1.108, *p* = 0.2967]. Likewise, on day 1 of Avoidance Extinction, groups were not different in Time Spent in the Shock Arm (Figure 2B; *p* = 0.5674) and Number of Shock Arm Entries (Figure 2D, *p* = 0.9065). These findings suggest that Cat Hair animals were able to learn fear conditioning and there was no impact on learning and expression of fear.

There was no effect of Cat Hair condition in Time Spent in the Shock Arm [Figure 2B; *F*(1,9) = 0.200, *p* = 0.6653, η^2^ = 0.068] and Number of Shock Arm Entries [Figure 2D; *F*(1,9) = 1.124, *p* = 0.3167, η^2^ = 0.152] indicating that both groups avoided the Shock Arm to a similar extent. Furthermore, there was an effect of Time on Time Spent in the Shock Arm [Figure 2B, *F*(3,27) = 8.078, *p* = 0.0005, η^2^ = 0.985] and Number of Shock Arm Entries [Figure 2D, *F*(3,27) = 9.112, *p* = 0.0002, η^2^ = 0.994] such that animals in both groups spent more time and made more entries in the Shock Arm over days of extinction. There was no interaction effect of Cat Hair condition × Time on Time Spent in the Shock Arm [*F*(3,27) = 0.272, *p* = 0.8452, η^2^ = 0.095] and on Number of Shock Arm Entries [*F*(3,27) =1.087, *p* = 0.3715, η^2^ = 0.254]. Combined, these findings suggest that both Cat Hair and Fake cat hair groups learned Avoidance Extinction/safety similarly well.

Although Cat Hair and Fake cat hair groups learned fear conditioning and fear extinction to the same extent (Figures 2B,D, Day 1), Cat Hair exposed animals made fewer entries over time and on Day 1 of extinction. There was an effect of Cat Hair condition on Total Entries [*F*(1,9) = 5.564, *p* = 0.0427, η^2^ = 0.553] as well as a significant difference on Day 1 (*p* = 0.0175) using *post hoc* tests. These findings suggests that cat hair exposure induced a long-term phenotypic difference. Despite this phenotypic difference, all animals had increased Total Entries over days of extinction as there was a main effect of Time on Total Entries [*F*(3,27) = 16.51, *p* < 0.0001, η^2^ = 1.000] with no Cat hair condition × Time interaction [*F*(3,27) = 1.116, *p* = 0.3599, η^2^ = 0.260]. Combined with the measures of extinction, these data suggest that cat hair exposed animals showed no deficits in learning but remained phenotypically different from controls 2 weeks after the mild stressor.

## Discussion

Anxiety and related anxiety-influenced disorders are sexually dimorphic with women being disproportionately affected compared to men. Given the increased prevalence in women and the documented differences in anxiety and trauma behavior between male and female rats (Shanazz et al., 2022) this paper sought to examine the link between stress, anxiety, and fear learning and extinction in female Sprague–Dawley rats. We tested the hypothesis that a mild stressor will have short-and long-term increases in anxiety and produce long term effects on subsequent fear learning and extinction behavior. We induced anxiety with a short exposure to a ball of cat hair (mild stressor) that elicits innate fear but does not cause fear conditioning (Vazdarjanova et al., 2001; Nalloor et al., 2011). The control group was exposed to fake cat hair. We found that cat hair exposure, as a stressor, induces changes in anxiety-like behavior in the short-and long-term without affecting fear learning and extinction.

Varying degrees of predator exposure has been known to elicit anxiety-like and defensive behavior in rats (Blanchard et al., 1986, 1989; Blanchard and Blanchard, 1989; Adamec and Shallow, 1993; Dielenberg and McGregor, 2001; McGregor et al., 2004; Muñoz-Abellán et al., 2009; Hacquemand et al., 2013). We have previously shown that cat hair exposure is equally stressful to male and female rats but that female rats expressed anxiety differently compared to males and engaged in anxioescapic behavior compared to males (Shanazz et al., 2022). Here we report that cat hair exposure induced short-term anxiety-like behavior that persisted for less than 4 days. This was illustrated by two tasks: EPM and LDOF (Shanazz et al., 2021) where cat hair exposed animals expressed more anxiety to open space than to light compared to controls. The anxiety phenotype of cat hair exposed animals included less exploration of the LDOF. Notably, these differences were not present before cat hair exposure demonstrating that there were no pre-existing differences between groups. The anxiety phenotype lasted beyond the initial stress response as demonstrated by their behavior on the EPM 24 h later where cat hair exposed animals made fewer Open Arm entries and had suppressed exploration with lower total arm entries. These results corroborate findings in male rats that a stressor induces anxiety-like behavior in the short-term (Blanchard et al., 1986, 1989; Blanchard and Blanchard, 1989; Adamec and Shallow, 1993; Dielenberg and McGregor, 2001; McGregor et al., 2004; Muñoz-Abellán et al., 2009; Nalloor et al., 2011; Hacquemand et al., 2013).

The duration of the short-term effect of the stressor on anxiety-like behavior was limited to 4 days as there were no differences on the LDOF 4 days after exposure. EPM was not tested again because it is subject to learning effects, unlike the LDOF (Shanazz et al., 2021). This finding is corroborated by previously published data showing that female rats tended to make an average of seven open arm entries on the EPM 5 days after cat hair exposure (Shanazz et al., 2022) which is similar to the number of entries made by the Fake cat hair exposed animals the day after exposure (average = 7). Together, these findings suggest that by 4 days after exposure their anxiety-like behavior is similar to baseline.

Despite this apparent resolution of the anxiety-like behavior there were long-term effects of the mild stressor as illustrated by changes in exploratory behavior to a novel space that was not previously paired with an aversive stimulus. Specifically, the cat hair exposed group had suppressed total entries in the Y-maze. Both groups spent the same amount of time exploring all arms of the maze indicating that the fewer entries in the cat hair exposed group did not affect their ability to explore a novel place. Suppressed exploratory movement was again demonstrated during the habituation phase of CFC where they made fewer crossings in the blocked off shock arm of the Y-maze. This cannot be explained by the fact that this was their second exposure to the Shock Arm because fake cat hair exposed animals did not show suppressed exploratory movement. The suppression effect was exaggerated by the size of the exploration environment such that it was more evident in a smaller environment. Cat hair exposed animals explored less when confined to the Shock Arm (exploration was ~18% of controls) than the entire Y-maze (exploration was ~54% of controls). To our knowledge, this is the first demonstration of a long-term effect of a mild stressor in adult rats. One study with female rats found that exposure to predator odor in adolescence resulted in reduced social interactions in adulthood (Wright et al., 2013) which could have been driven by long term developmental changes in anxiety which is documented in rats (Avital and Richter-Levin, 2005).

Another long-term change induced by the mild stressor exposure is a phenotypic change that does not affect learning of fear association or fear extinction. The phenotypic difference was evident in overall activity in the Y-maze where cat hair exposed animals had significantly fewer total entries. This did not have an effect on learning and memory as there was no difference in avoidance extinction between cat hair and fake cat hair exposed animals. Combined, these data suggest that the mild stressor may be affecting some generalized anxiety-like behavior independent of learning of safety in the long-term. It may also suggest that cat hair exposure facilitates stronger generalization of fear to environments that were not paired with the foot-shock but were previously associated with the foot-shock paired context, i.e., the safe arms.

The existence of both short-and long-term effects on anxiety-like and exploratory behavior highlights an important consideration that the impact of stress, even a mild stressor, might have subtle impacts on behaviors of interest. It remains to be determined what the implications of these behavioral observations and their relationship with neuronal circuit function are. We have previously shown that male rats in a negative emotional state impacts the encoding of ongoing emotionally neutral events in the hippocampus (Dixon-Melvin et al., 2022). From a mechanistic perspective, it is possible that the long-term effects of the stressor on the rats have altered their state sufficiently such that when exploring a novel environment, they are perceiving it as more stressful than control rats and thus, although they appear to extinguish fear responses similarly to fake cat hair exposed animals, they are in fact having an entirely different experience. These effects have been previously demonstrated with trauma paradigms with only a week between the stressor and the foot-shock (Nalloor et al., 2011, 2014; Dixon-Melvin et al., 2022). In future experiments we will examine if these findings are sexually dimorphic.

Anxiety effects from predator stress exposure have been shown to have strain dependent effects (Cohen et al., 2006) such that Lewis rats tend to have more extreme anxiety-like responses compared to Sprague–Dawley. Moreover, it is known that stress enhances fear learning (Izquierdo et al., 2016). The lack of differences in learning of safety in the long-term in the current study may be an effect of strain and a prolonged period (2 weeks) between the mild stressor exposure and CFC testing. Additionally, the mild stressor was not of a traumatic nature as the parameters used in this experiment do not induce CFC comparable to foot-shock. This may also explain why there are no long-term effects on learning of safety. Therefore, a mild stressor exposure 2 weeks before a traumatic event does not necessarily have any effect on PTSD-like behavior in female Sprague–Dawley rats but it does generate phenotypic differences in how those previously stressed rats behave in a traumatic context.

Despite the above-mentioned limitations, this work has some implications for observations in humans. It is well documented that people in high stress situations, where they are likely to encounter traumatic events such as public safety and fire fighters (Carlier et al., 1998; Marmar et al., 2006; Pinto et al., 2015), military service (Bleich and Solomon, 2004; Berge et al., 2020), and other chaotic environments (Breslau et al., 1999; Gillespie et al., 2009; Shalev et al., 2019) have a higher probability of developing PTSD. While our findings do not directly indicate that PTSD-like behavior is exacerbated by the mild stressor exposure, their susceptibility status might be changed in the long-term by previous stress exposure as indicated by the existence of phenotypic differences due to cat hair exposure. A further stressor/traumatic event could result in PTSD-like behavior. Future studies will examine the effect of repeated mild stressor exposure on long-term anxiety-and PTSD-like behavior following a subsequent traumatic stressor. Nonetheless, our behavioral findings in these rats indicate that even a mild stressor has a long-term impact on behavioral interaction with the environment.

The current findings also suggest broader implications for research with animals with the caveat that this study was done in female rats. The subtle impact of the mild stressor on long term behavior highlights the importance of considering all conditions which may be stressful such as housing, handling, and sequence of behavioral testing as it may affect reproducibility.

## Data availability statement

The raw data supporting the conclusions of this article will be made available by the authors, without undue reservation.

## Ethics statement

The animal study was approved by Institutional Animal Care and Use Committee (IACUC) at the Charlie Norwood VA Medical Center (CNVAMC). The study was conducted in accordance with the local legislation and institutional requirements.

## Author contributions

KS and AV wrote the manuscript with input from RN. KS, AV, and RN participated in the designing and running of experiments. KS performed behavioral testing and data acquisition. All authors contributed to the article and approved the submitted version.

## Funding

This material is based upon work supported in part by the Department of Veterans Affairs, Veterans Health Administration, Office of Research and Development, Biomedical Laboratory Research and Development, I01BX001978 and I01BX003890.

## Conflict of interest

The authors declare that the research was conducted in the absence of any commercial or financial relationships that could be construed as a potential conflict of interest.

## Publisher’s note

All claims expressed in this article are solely those of the authors and do not necessarily represent those of their affiliated organizations, or those of the publisher, the editors and the reviewers. Any product that may be evaluated in this article, or claim that may be made by its manufacturer, is not guaranteed or endorsed by the publisher.
