# Supplementary material for: A mild stressor induces short-term anxiety and long-term phenotypic changes in trauma-related behavior in female rats
Source: Front Behav Neurosci. 2023 Sep 4;17:1231563. doi: 10.3389/fnbeh.2023.1231563 (PMC10507355; doi:10.3389/fnbeh.2023.1231563)
Supplement: Supplementary file 1 [file Data_Sheet_1.pdf]

Supplementary Material

A mild stressor induces short-term anxiety and long-term phenotypic changes in trauma-related behavior in female rats

Khadijah Shanazz, Rebecca Nalloor, and Almira Vazdarjanova*
***Correspondence:** Almira Vazdarjanova: avazdarjanova@augusta.edu

Supplementary Methods:

Female Sprague-Dawley Rats (n= 24) were tested in the LDOF. The next day they were exposed to cat hair and 24 hours later their contextual fear conditioning was assessed by measuring freezing behavior in an identical box which did not contain cat hair. Freezing was defined as the lack of movement except that related to breathing.

Five days after cat hair exposure they were tested for acoustic startle response. The next day, they were tested for anxiety-like behavior on the elevated plus maze. The following day they received foot-shock/context pairing in the isolated Shock Arm of the Y-Maze. Twenty-four hours later their freezing was assessed in the isolated Shock Arm. Specific test parameters were the same as those described in detail in the Methods section of the main article, except for the 24-hour contextual fear conditioning test which in this group was done in the Shock Arm isolated from the rest of the maze, while in the main article it was open to the other two arms.

Supplementary Figure:

Freezing at 24hrs post exposure

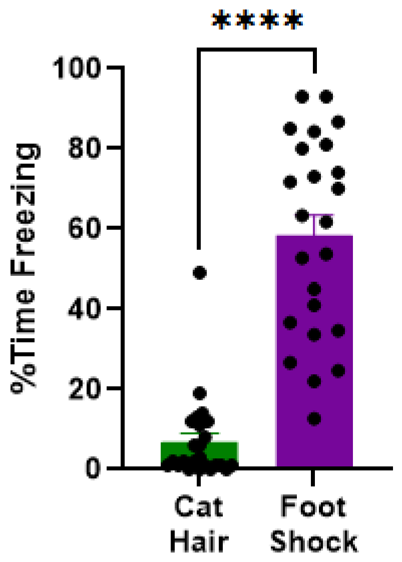

Supplementary Figure 1. Percent time spent freezing 24 hours after cat hair or foot-shock exposure. ****$p<0.0001$, Repeated Measures ANOVA.

Animals spent more time freezing in a context identical to that during exposure24 hours after foot-shock/context compared to cat hair/context pairing (Repeated Measures ANOVA: $F(1,23)=117.484$, $p < 0.0001$, $\eta= 1.000$). This finding indicates that cat hair is a mild stressor compared to foot-shock which induces contextual fear conditioning.
